# Finding the mean in a partition distribution

**DOI:** 10.1186/s12859-018-2359-z

**Published:** 2018-10-12

**Authors:** Thomas J. Glassen, Timo von Oertzen, Dmitry A. Konovalov

**Affiliations:** 1 Department of Psychology, Universität der Bundeswehr München, Werner-Heisenberg-Weg 39, Neubiberg, 85577 Germany; 2 Max Planck Institute for Human Development, Department for Lifespan Psychology, Berlin, Lentzeallee 94, Berlin, 14195 Germany; 3 School of Information Technology, James Cook University, 1 James Cook Drive, Townsville, QLD 4811 Australia

**Keywords:** Mean partition, Partition distance, Bayesian clustering, Dirichlet Process

## Abstract

**Background:**

Bayesian clustering algorithms, in particular those utilizing Dirichlet Processes (DP), return a sample of the posterior distribution of partitions of a set. However, in many applied cases a single clustering solution is desired, requiring a ’best’ partition to be created from the posterior sample. It is an open research question which solution should be recommended in which situation. However, one such candidate is the sample mean, defined as the clustering with minimal squared distance to all partitions in the posterior sample, weighted by their probability. In this article, we review an algorithm that approximates this sample mean by using the Hungarian Method to compute the distance between partitions. This algorithm leaves room for further processing acceleration.

**Results:**

We highlight a faster variant of the partition distance reduction that leads to a runtime complexity that is up to two orders of magnitude lower than the standard variant. We suggest two further improvements: The first is deterministic and based on an adapted dynamical version of the Hungarian Algorithm, which achieves another runtime decrease of at least one order of magnitude. The second improvement is theoretical and uses Monte Carlo techniques and the dynamic matrix inverse. Thereby we further reduce the runtime complexity by nearly the square root of one order of magnitude.

**Conclusions:**

Overall this results in a new mean partition algorithm with an acceleration factor reaching beyond that of the present algorithm by the size of the partitions. The new algorithm is implemented in Java and available on GitHub (Glassen, Mean Partition, 2018).

## Background

### Introduction

*Structurama* [1, 2] is a frequently used software package for inferring the population structure of individuals by genetic information. Despite the popularity of the procedure [3], high computational costs are frequently mentioned as practical limitations [4, 5]. The tool uses a DP mixture model (DPMM) and an approximation method to determine the mean of the generated samples. This mean can be viewed as the expected clustering of the DPMM if the number of considered samples approaches infinity.

Because the approximation method can significantly contribute to the required computation time of Structurama, we develop two optimized variants in this article. In doing this, we intentionally refrain from reducing the calculation effort by taking a competely different, but more light-weight approach. For example, one could use Variational Bayes instead of Markov chain Monte Carlo (MCMC) Sampling or replace the mean partition approximation by an alternative consensus clustering algorithm (e.g., CC-Pivot [6]). Both strategies lead to faster procedures relatively easily, but in many cases the accuracy of the calculated means can be severely impaired. This applies to both MCMC versus Variational Bayes [7] and mean partition approximation versus other consensus clustering approaches [8, 9]. In contrast, our resulting algorithm offers the same accuracy as the original method at a significantly lower runtime complexity.

Furthermore, our achieved runtime complexity also represents a significant advance with respect to other consensus clustering methods based on the mean partition approach. To the best of our knowledge, no other method with this approach has been published to date with only a linear factor *N* in its runtime complexity. Previous variants, also known as local-search procedures, could not undercut a factor of *N*^2^ and are therefore considered impracticable for realistic datasets [8–11]. Our resulting method achieves such a linear factor *N* and thus enables accurate and fast calculations of a consensus for multiple clustering results.

Below we first describe the original algorithm. We then present our improvements, followed by a detailed benchmark of the resulting method.

### Mean partition approximation algorithm

The considered algorithm for approximating the mean partition begins by choosing any initial clustering. Afterwards it iterates through all *N* individuals *p*_*i*_ and all *C* existing clusters *c*_*j*_, including one empty cluster, and checks whether the movement of *p*_*i*_ to *c*_*j*_ improves the solution. If that is the case, *p*_*i*_ is re-assigned to cluster *c*_*j*_, else it stays in its old cluster. The process is repeated until no changes occur in a full cycle through all individuals and clusters [1, 12]. To check whether the solution improves, the algorithm computes the distance between the candidate solution and all partitions *K* in the posterior sample. The number of distance measures therefore equals the number of individuals *N* times the number of clusters *C* in the candidate solution times the number *K* of partitions. In summary, this results in *O*(*NCK*) distance measures.

A naive method for computing the distance is very time intensive. For example, the first (recursive) algorithm suggested by [13] has an exponential runtime [14]. Therefore, a common solution (e.g. [12]) is to compute the distance between two partitions by executing the following two steps. First, the problem is reduced to a linear sum assignment problem (LSAP) via the procedure of [15] in *O*(*NC*^2^). Afterwards, the Hungarian Algorithm is applied [16, 17], which requires *O*(*C*^3^) steps. In total, this approach results in *O*(*N*^2^*C*^3^*K*) steps per cycle by the current mean partition approximation algorithm.

Next we briefly desribe the reduction of [15], introduce a faster alternative, and give an overview of the Hungarian Method.

### Reduction of the partition distance problem to the LSAP

Konovalov et al. [15] discovered that the partition distance matches the minimum costs of the solution of a LSAP and established the reduction

1 $$  D(A,B) = \text{min}\sum\limits_{ij} x_{ij}|a_{i} \cap \bar b_{j}|  $$

Here $|a_{i} \cap \bar b_{j}|$ corresponds to the entry *c*(*i*,*j*) of the cost matrix for the LSAP. *a*_*i*_ and *b*_*j*_ denote strings of bits that represent the *N* elements of the partitions *A* and *B* and are set to 1 if their associated elements belong to cluster *i* and *j*, respectively. *x*_*ij*_ describes additional assignment-limiting variables, so that each cluster *i* is assigned to exactly one cluster *j* and vice versa. The selection of these *x*_*ij*_, with the goal of minimizing the sum, is essentially the aim of the Hungarian Method. To build up a cost matrix for the latter, a direct algorithmic transfer of (1) would obviously lead to a runtime complexity of *O*(*NC*^2^). This is because the bit strings have the length *N*. The reduction complexity of this approach is therefore nearly always higher than that of the optimal Hungarian Algorithm, which has a runtime of *O*(*C*^3^).


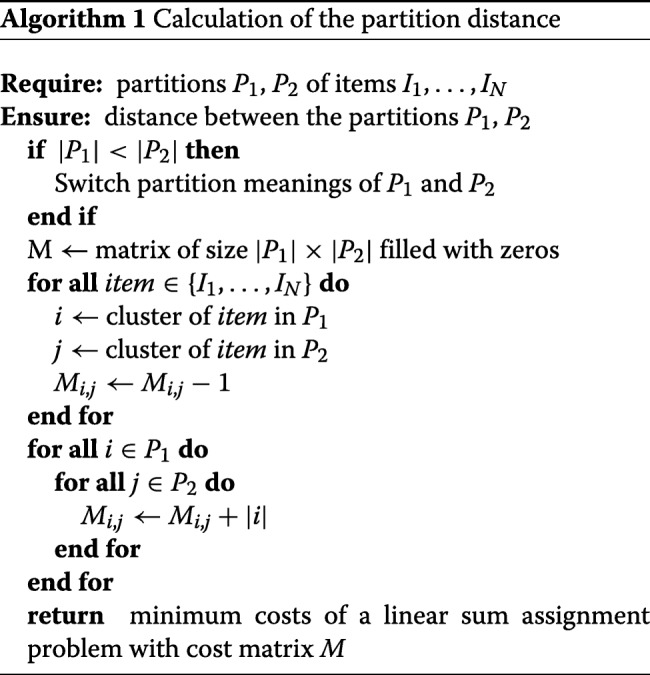


Indeed the direct algorithmic transfer seems to be the typical implementation, as has been observed in source codes reviewed so far by the first author, e. g. in [15] or [12]. We would therefore like to draw attention to a faster variant, which only needs *O*(*C*^2^+*N*) for the same reduction and thus reduces the partition distance calculation from *O*(*NC*^2^) to *O*(*C*^3^+*N*). It is the procedure of [18], which is shown in Algorithm 1. The runtime reduction is achieved by ignoring the stated calculation in (1) and accounting for its meaning instead. Thus, (1) says that the cells *c*(*i*,*j*) of the *C*×*C* cost matrix for the Hungarian Algorithm have to keep the number of those elements of the cluster *i*∈*P*_1_, which are not contained in cluster *j*∈*P*_2_. We can therefore construct the matrix faster by first billing for each element a distance reduction of 1 for that single cluster pair, which has this element in common. Subsequently, we add the size of each cluster *i* to each cell *c*(*i*,*j*).

Using this reduction, the new cycle runtime of the whole mean partition approximation algorithm is *O*(*NC*^4^*K*+*N*^2^*CK*) instead of *O*(*N*^2^*C*^3^*K*). That means, that now both complexities correspond solely if the number of clusters *C* equals the number of elements *N* in the partitions. In a typical scenario, however, we have *C*<<*N*. For example, we can expect *α* log*N* clusters for a DP-based grouping of observations [19]. In such cases the new reduction leads to a complexity decrease of two orders of magnitude because *N*^2^*CK* dominates its term.

### Hungarian Algorithm

We now briefly review the Hungarian Algorithm, which assigns *C* workers to *C* jobs (assigning an unequal number of workers to jobs can be done with simple adaptations). Let *c*(*i*,*j*) be the costs if worker *i* does job *j*. The Hungarian Algorithm works on a directed bipartite graph *G*=(*S*,*T*;*E*) where one node set consists of the workers and the other node set consists of the jobs. The edges *E* change in the steps *k*=1,... of the Hungarian Algorithm. In addition, a function $y: S \cup T \rightarrow \mathbb {R}$, called the *potential*, labels the nodes with numbers.

At every step, the following invariants are upheld: (1) every node is adjacent to at most one edge from *T* to *S*, (2) for every edge (*i*,*j*)∈*E* in either direction, we have *y*(*i*)+*y*(*j*)=*c*(*i*,*j*), and (3) for every pair (*i*,*j*), we have *y*(*i*)+*y*(*j*)≤*c*(*i*,*j*). Let *M* be the subgraph of all edges from *T* to *S*; *M* implies a matching of its nodes. It can be proven that, if all nodes of *S* are included in *M*, *M* is a solution to the job assignment problem on its nodes (for further details see [20]). Initially, *y*(*i*)=0 for all nodes, and *E* contains exactly those edges (*i*,*j*) with *c*(*i*,*j*)=0, directed from *S* to *T*. Note that the invariants above are satisfied. At every step, we find all nodes in *S* and *T* that are reachable from nodes in *S* not already in *M*. Let us call these *S*_*reach*_ and *T*_*reach*_. If a node of *T* is reachable and not already in *M*, the direction of all edges on this path are reversed. Obviously, this increases the number of edges in *M* by one. Otherwise,let

2 $$ \Delta = \min\{c(i,j) - y(i) - y(j) | (i,j) \in S_{reach} \times T / T_{reach}\}  $$

which corresponds to the minimum costs minus the potential from reachable nodes in *S* to non-reachable nodes in *T*. We then increase *y*(*i*) by *Δ* for all nodes *i*∈*S*_*reach*_ and reduce *y*(*j*) by *Δ* for all nodes *j*∈*T*_*reach*_. Note that in this way, the invariant is still satisfied, but edges are added and removed from *G*. By design, the reachability of the non-matched nodes increases by at least one further node in *T*. As soon as *M* includes all nodes of *S*, we stop and return the matching implied by *M*. Note that the total costs of the matching is the sum of all *y* values, ${\sum \nolimits }_{i \in S \cup T} y(i)$. The Hungarian Algorithm requires *O*(*C*^4^) steps in the version presented here, but adaptations exist [21] that work in *O*(*C*^3^) steps.

## Methods

### Improvement of the approximation algorithm

In the current mean partition approximation algorithm, we need to carry out a problem reduction to a LSAP for each distance calculation between two partitions. However, as we have previously noted, the reduction can often be more expensive than the Hungarian method itself. In addition, there is only a minimal distance change between each sample partition and the candidate partition when an individual is moved into another cluster. We can therefore speed up the process by maintaining and dynamically adjusting a bipartite graph *G* for each pair of sample- and candidate partitions.

Let *P*_1_ be a sample partition and *P*_2_ the candidate partition, which we optimize step by step. We declare *C*_1_ as the cluster of an individual *p* in *P*_1_ and *C*_2_ as the match of *C*_1_ in *P*_2_. In addition, *E*_2_ denotes the cluster of *p* in *P*_2_ and *D*_2_ the cluster into which *p* will be moved.

We recognize that if we move an individual *p* from cluster *E*_2_ with index *j* to the cluster *D*_2_ with index *k*, only the row *i* of the cost matrix associated with cluster *C*_1_ changes. Thus, *c*(*i*,*j*) becomes more expensive by one after the removal of *p*, and *c*(*i*,*k*) reduces by one after adding *p*. To keep the condition *y*(*i*)+*y*(*j*)≤*c*(*i*,*j*) satisfied, we will not reduce costs in a cell. We leave them unchanged instead, increase every other cell in the row by one and substract one from the final distance. In summary, we increment *c*(*i*,*j*) as well as every cell of the row except *c*(*i*,*k*). Then we remove the matching edge of *C*_1_ and *C*_2_ from *M* and carry out another step in the Hungarian Algorithm to rematch *C*_1_. This step costs *O*(*C*^2^). Finally, we decrement the result by one.

If a movement of *p* does not lead to a better distance, we have to restore the last best state of the graph *G* for *P*_1_ and *P*_2_ before continuing with the next *p*. Saving and restoring the state of the graph is feasible in *O*(*C*), since only one row of the cost matrix changes. A cycle of the improved approximation algorithm runs in *O*(*NC*^3^*K*) and is therefore at least one order of magnitude faster then the original version with *O*(*NC*^4^*K*+*N*^2^*CK*). The new procedure is shown in Algorithm 2.


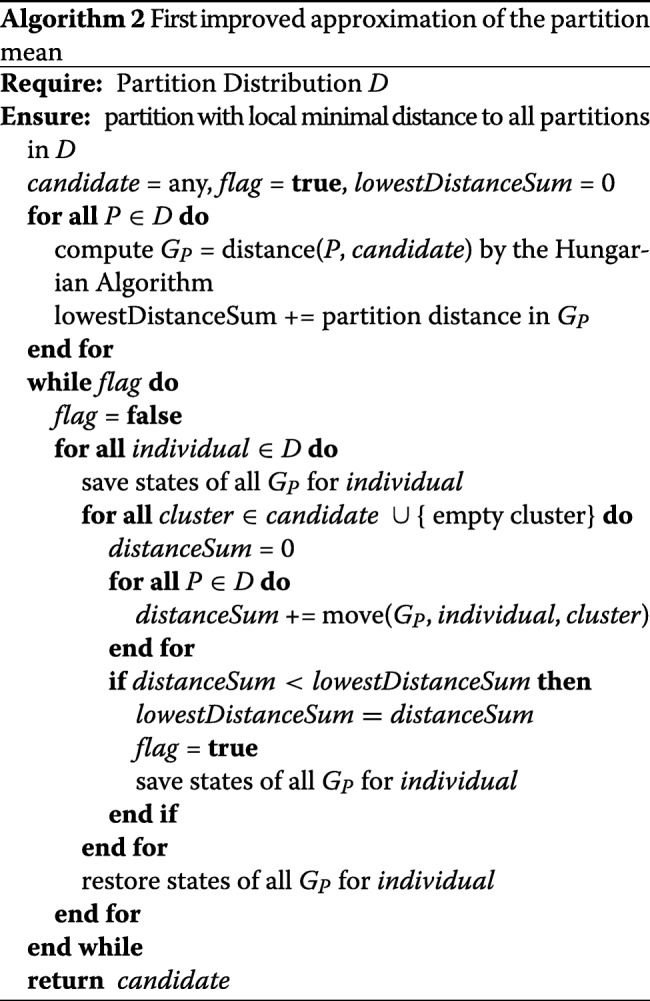


In the next subsection we show that the new time complexity is still not at its optimum. We proceed by describing a detailed theoretical procedure to reduce it further.

### Further improvement

To achieve this, we have to reduce the costs of performing a step in the Hungarian Algorithm, which takes *O*(*C*^2^) steps so far. These costs are essentially caused by a Breath First Search or Depth First Search (BFS/DFS), which is necessary if the reachability of a node from another node has to be queried. The cost is due to the fact that the bipartite graph has a maximum of *C*^2^ edges, all of which must be tested in the worst case. Thus, in order to improve the approximation further, the reachability check has to be done with costs <*O*(*C*^2^). A first approach would be to calculate the reachability between all node pairs beforehand, that is, before considering the movement for each individual *p*. Then we could query the reachability within the local cluster optimization loop in *O*(1).

Calculating the All-Pairs Shortest-Paths (APSP) via the classical methods Floyd-Warshall or Dijkstra is ruled out. This is because the former requires *O*(*C*^3^) and the latter has the same costs in the case of *C*^2^ edges. Using one of these algorithms would result in the same time complexity as our first improved algorithm, because we have to run them before the local cluster optimization loop for each iteration of *p*. In summary, we need the All-Pairs-Reachability (APR) per *p* with guaranteed pre-calculation costs <*O*(*C*^3^) and query costs <*O*(*C*^2^).

In principle, three approaches are conceivable. These are dynamic APSP methods and static and dynamic APR procedures. Dynamic methods have the advantage that updates are cheaper than a complete recalculation. For example using the method of [22], we can delete or add any number of incoming edges of/to a node and subsequently update the APSP with amortized costs in *O*(*C*^2^ log*C*). In principle, the cost of a new calculation of *O*(*C*^3^) is then surpassed, but unfortunately the method is still not suitable here. The reason for this is the worst case number of needed updates if a path has to be reversed after the movement of an individual *p*. For example, a path can consist of 2*C*−1 edges and cover 2*C* vertices. In this worst case, we have to carry out *C* updates with the method of [22]. The amortized update costs therefore increase to *O*(*C*^3^ log*C*), which is worse than a recalculation with Floyd-Warshall. Static APR methods are currently not suitable for our situation either. This is because current methods require properties of the graph for a sub-cubic runtime, which are not present in our case (for an overview, see [23]). Finally, we have the dynamic APR methods, from which the deterministic variants suffer from a comparable update problem as the dynamic APSP methods. Among Monte Carlo methods, however, there is a variant that is ideally suited for our situation. It is the method of [24], which dynamically calculates the transitive closure via the dynamic matrix inverse. A requirement of this approach is a graph with perfect matching. This method updates one edge of the graph and its transitive closure in *O*(*C*^1.575^) and queries the reachability in *O*(*C*^0.575^). Thus, if we have a worst case scenario and want to carry out 2*C*−1 edge updates, we now have total costs of *O*(*C*^2.575^). This is cheaper than *O*(*C*^3^) by a factor of almost $\sqrt C$ and reduces the final cost of a cycle from *O*(*NC*^3^*K*) to *O*(*NC*^2.575^*K*).

We now want to be able to replace a single step in the Hungarian Algorithm only by operations in constant time and reachability queries in *O*(*C*^0.575^). To achieve this, we first need to distinguish two situations when moving an individual *p* from cluster *E*_2_ to cluster *D*_2_. For both, we examine the present graph and (if possible) directly determine the costs after the movement.

**Case A**: *E*_2_≠*C*_2_

If *D*_2_ equals *C*_2_, the matching of *C*_1_ and *C*_2_ becomes cheaper and the cost is reduced by one. Otherwise, if there is an edge from *C*_1_ to *D*_2_ and *C*_2_ is reachable from *D*_2_, the cost is also reduced by one. This is because only the costs between *C*_1_ and *D*_2_ remain constant. Thus, if there was already an edge (*C*_1_,*D*_2_) and a path from *D*_2_ to *C*_2_, we can reverse this path and the edge (*C*_1_,*D*_2_) with no additional costs. The final cost reduction of one then reduces the costs by one. If neither of the two situations is given, we have a cost change of zero.

**Case B**: *E*_2_=*C*_2_

Similar to the second sub-case of case *A*, we have a cost reduction of one, if there is an edge (*C*_1_,*D*_2_) and a path from *D*_2_ to *C*_2_. If this is not the case, we can at least get the same costs if there is any path from *C*_1_ to *C*_2_. An alternative for equal costs exists when the second sub-case of case *A* can be achieved with a potential increase of one. In this case, the final cost reduction leads to a cost change of zero. Any other non-considered situation will result in a cost increase by one.

Within the cluster optimization loop, we can directly examine case *A* with a worst case cost of *O*(*C*^0.575^). The latter is due to the possibility of a needed reachability check. For case *B*, on the other hand, we possibly need APSP in the last sub-case. This is because we ask for the existence of paths that require a maximum potential increase of one. The method of [24], however, only provides reachability checks via adjacency matrices. Therefore, we have to handle this last sub-case separately. We divide it as follows:

**Case B.3.1**: (*C*_1_,*D*_2_) exists, there is a path from *D*_2_ to *C*_2_

This situation has already been dealt within the first sub-case of case *B*. It leads to a cost reduction of one.

**Case B.3.2**: (*C*_1_,*D*_2_) exists, there is no path from *D*_2_ to *C*_2_

If a path exists after a potential increase by one for a worker of the graph, then the cost remains the same. Otherwise, the movement of *p* leads to a cost increase by one.

**Case B.3.3**: (*C*_1_,*D*_2_) exists after a potential increase by one, there is a path from *D*_2_ to *C*_2_

We have a path from *C*_1_ to *C*_2_ via *D*_2_ that requires a potential increase of one at most. This means that the costs remain the same.

**Case B.3.4**: (*C*_1_,*D*_2_) exists after a potential increase by one, there is no path from *D*_2_ to *C*_2_

For a path from *D*_2_ to *C*_2_, a potential increase for a worker of the graph is required. However, we already have the maximum increase of one, which means that the costs increase by one.

**Case B.3.5**: (*C*_1_,*D*_2_) exists after a potential increase by more than one

In this situation, the costs increase by one.

Except for the B.3.2 sub-case, all other sub-cases can be tested via reachability checks in *O*(*C*^0.575^). However, if *B*.3.2 occurs, there is another situation in which we do not need to know whether there is a path after a potential increase or not. The presence of this situation is indicated by the current cost change *CC*_*best*_ by the remaining of the *K* graphs in which *B*.3.2 does not occur. If *CC*_*best*_ is greater or equal to the current best local change *L*_*best*_, the movement of *p* is definitely more or equally expensive, since the *B*.3.2 sub-cases can not reduce costs. Therefore, we do not need to evaluate these graphs in which *B*.3.2 is present, but continue with the next cluster or the next individual.

If, on the other hand, *CC*_*best*_<*L*_*best*_, then we must decide some or all of these graphs with *B*.3.2 with higher computational effort. To do this, we first select one of the undecided graphs and evaluate it using the method presented below. We then update *CC*_*best*_ with the cost change by this graph. If *CC*_*best*_ becomes ≥*L*_*best*_, we will terminate and continue with the next cluster or the next individual. In the worst case, we must decide all graphs with *B*.3.2 with higher computational effort.

The evaluation as to whether the cost change is one or zero in a graph with sub-case *B*.3.2 works as follows: We collect all reachable workers and jobs for *D*_2_ and *C*_2_ in *O*(*C*^1.575^). We then assume that we have access to a row- and column-sorted *C*×*C* matrix, whose cells store the values *c*(*i*,*j*)−*y*(*i*)−*y*(*j*) for each worker-job pair (the so-called slack values). How and when this matrix is constructed is explained later. Since we have this matrix, we sort the reachable workers and jobs from both *D*_2_ and *C*_2_ according to their row and column numbers in the slack value matrix in *O*(*C* log*C*). Then we look for a slack value of one in the sub-matrix for the workers of *D*_2_ and the jobs of *C*_2_ as well as in the sub-matrix for the jobs of *D*_2_ and the workers of *C*_2_ in *O*(*C*) using Saddleback Search [25]. If this value has been found in one of the two sub-matrices, the costs remain the same, otherwise they increase by one. Note that a value of zero can not be present, since case *B*.3.2 would not have occurred otherwise. The total runtime of this procedure is *O*(*C*^1.575^) and is thus smaller than a BFS / DFS with *O*(*C*^2^).

An unsorted slack value matrix can be constructed in *O*(*C*^2^) and sorted in rows and columns in *O*(*C*^2^ log*C*). We calculate it initially and whenever we update the transitive closure.

### Resulting Algorithm

In contrast to the first improvement, we will neither store the states of the graphs before continuing with the next individual *p* nor will we restore them at any point. Instead, we initially calculate the adjacency and slack value matrix in *O*(*C*^2^ log*C*) and prepare the transitive closure matrices according to [24] in *O*(*C*^2.376^). In the local cluster optimization loop, we then determine the new costs in *O*(*C*^1.575^) and memorize the best cluster movement. After each local optimization, we only update each graph, the corresponding adjacency matrix, and its transitive closure in *O*(*C*^2.575^) if a better cluster was found for *p*. By doing this, not only the worst-case performance is reduced to *O*(*NC*^2.575^*K*) per cycle, but also the costs for the best case. The latter is given when we do not have to deal with the sub-case *B*.3.2 in a cycle. In such a situation the runtime drops from *O*(*NC*^3^*K*) to *O*(*NC*^1.575^*K*). On average, the second improvement is therefore clearly faster than *O*(*NC*^2.575^*K*) per cycle. The procedure is shown in Algorithm 3.


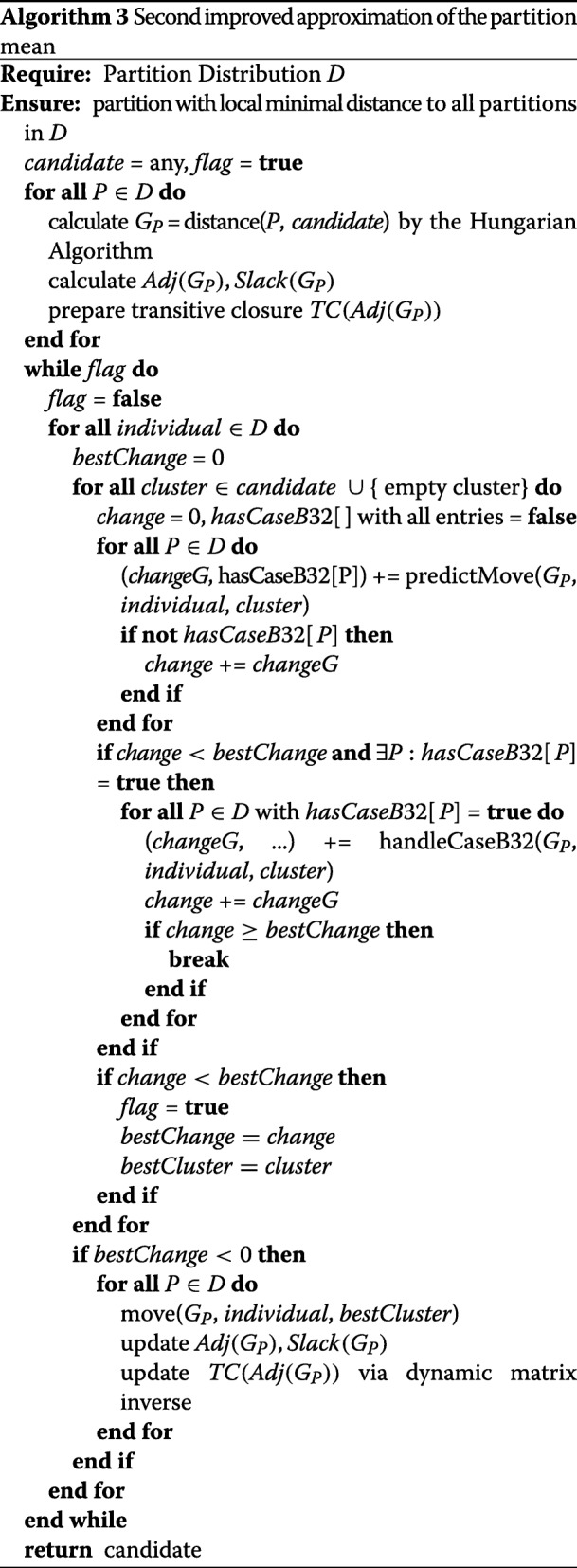


Note that this last improvement is currently only theoretical. This is the case, because the dynamic transitive closure of [24] is based on fast matrix multiplication via the Coppersmith-Winograd algorithm [26]. For the latter there is currently no feasable implementation, since its benefit would only arise for matrices too large for practical purposes [27, 28]. However we are confident that this improvement can be of practical value in the future, if similar developments are achieved for the Coppersmith-Winograd algorithm as for the preceding Strassen algorithm. The latter, also regarded as impractical initially, nowadays has feasable implementations [29].

In the next section, we will have a closer look at the faster reduction of [18] and the first improvement of the approximation algorithm. For both we will give benchmark results and compare them to their original counterparts.

## Results

### Comparison of old and new partition distance calculation

**Fig. 1:**
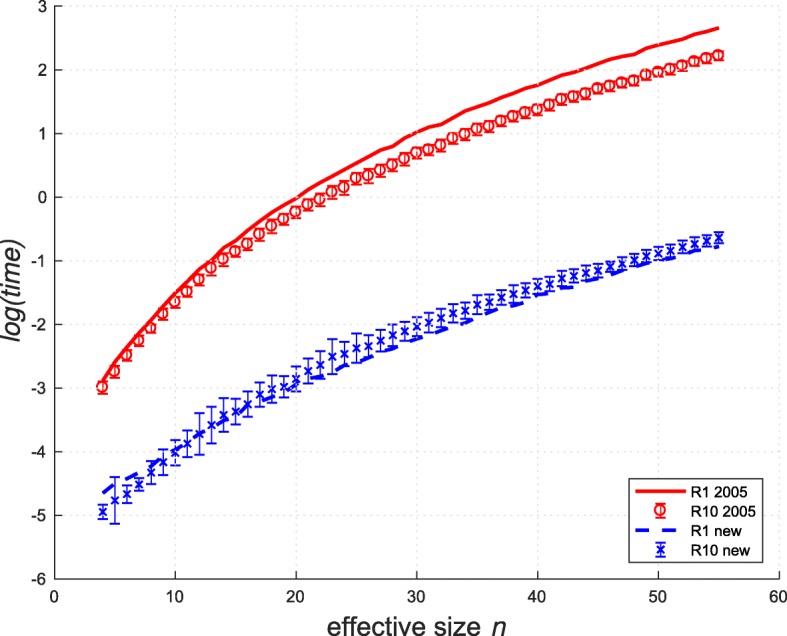
Performance comparison of time taken to calculate partition-distance on the R1 and R10 simulation sets. Natural logarithm of time is displayed in arbitrary units against the effective partition size *n*. Each point is an average over 100 different distance calculations, where the error bars show one standard deviation for R10 test

**Fig. 2:**
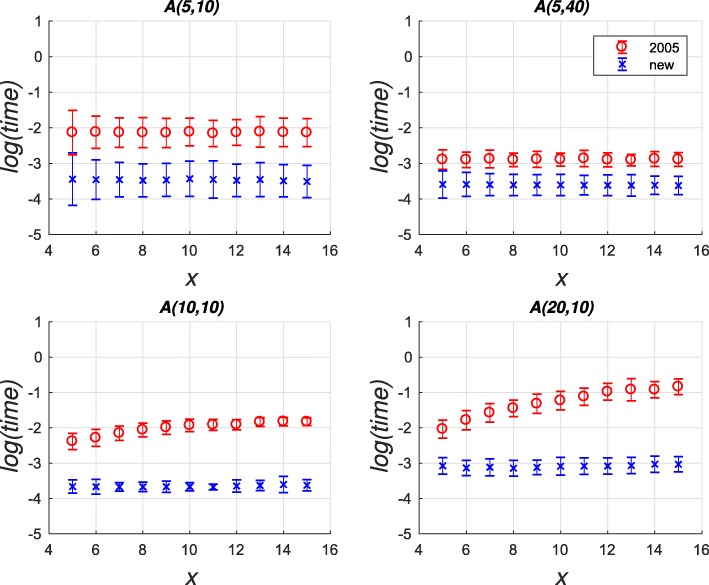
The same performance comparison as in Fig. 1 but for the simulation set RM. Natural logarithm of time is displayed in arbitrary units against the number of randomly moved individuals *x*

When assessing the population structure reconstruction results, statistical simulation is the preferred approach, since the simulation provides the ground-truth target partitions. When comparing the reconstruction and the target partitions, the partition distance is the most common metric of accuracy. The distance is defined [13] as the minimum number of individuals that need to be removed from each partition in order to leave the remaining partitions equal. To compare our calculation approach of the partition distance with the new reduction against the original Java-based partition-distance algorithm (denoted by “2005” in Figs. 1 and 2) of [15], we implemented Algorithm 1 in Java and used the Java-based implementation of the Hungarian Method of [30] to solve the LSAP. In Fig. 1, the R1 and R10 simulation tests reproduced the kinship assignment testing in [31] and [32] for the extreme case of samples containing only unrelated individuals and each group containing 10 individuals, respectively. See [15] for the detailed description of the tests. The new approach achieves an improvement of two orders of magnitude for most effective partition sizes *n*. For two given partitions *P*_1_ and *P*_2_, we defined the latter as *n*= max{*C*_1_,*C*_2_} after all identical clusters are removed.

The RM simulation test [15] is presented in Fig. 2, where each sub-figure title *A*(*C*,*M*) denotes *C* clusters, each containing *M* individuals. To simulate the misclassification errors of a reconstruction algorithm, we created partition *P*_2_ by randomly moving *x* individuals to a different cluster. The new approach was consistently faster and did not exhibit deterioration in speed when the partition distance increased with growing *x*.

### Comparison of old and new mean partition calculation

In the following, the improved partition distance reduction was used both for the old and the new mean partition algorithm [33]. In this way we show that the new algorithm alone already represents a significant improvement.

First, we analyze the influence of the partition size on the computation time. Here, we used the famous Iris Flower Dataset (IFD) of [34] as a comparison benchmark. To receive different partition sizes in our posterior sample, we expanded the IFD stepwise by new individuals drawn from assumed cluster-specific multivariate Gaussian distributions. Besides the original size of the sample of 150 individuals with 50 individuals per cluster, data sets with 250 to 1050 individuals per cluster were generated in this way.

For every dataset we first drew 100 partitions from a DP Gaussian mixture model (DPGMM) and then determined their mean partition using the old and new algorithm. When the dataset contained < 850 individuals per cluster, the mean partitions consistently showed a two-cluster solution. Only the two largest datasets revealed the actual partition structure with three clusters.

**Fig. 3:**
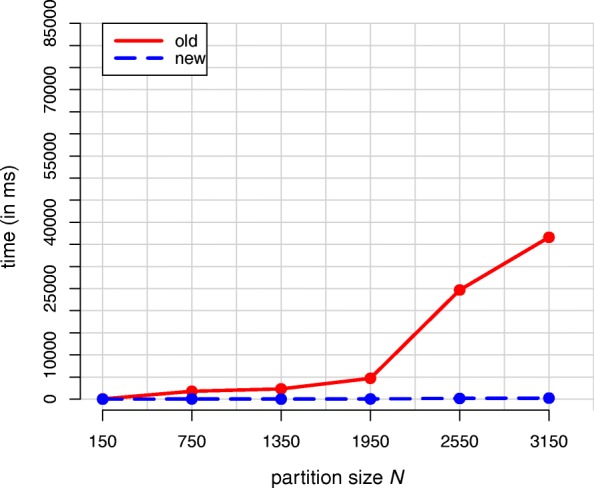
Average effort of 100 calculation repetitions using the old and the new mean partition algorithm as a function of the partition size *N*

Figure 3 shows the course of the average calculation effort of 100 calculation repetitions for different partition sizes *N* and both algorithms. All computations were carried out on a laptop with i7-4870HQ CPU. Table 1 lists the corresponding calculation times in milliseconds, as well as the acceleration factor of the new versus the old variant.

**Table 1:** Average calculation time (in ms) of 100 calculation repetitions for different partition sizes

*N*	Time (old)	Time (new)	Factor
150	44.9 (± 0.2)	4.0 (± 0.1)	11.2
750	1783.3 (± 2.1)	42.7 (± 0.3)	41.8
1350	2318.2 (± 2.0)	33.2 (± 0.3)	69.8
1950	4706.0 (± 3.2)	47.1 (± 0.3)	100.0
2550^a^	24681.0 (± 47.5)	181.3 (± 0.9)	136.1
3150^a^	36614.3 (± 17.0)	222.0 (± 1.0)	164.9

The clustering-samples of the DPGMM for the datsets marked with ^a^ suggested a three- instead of two-cluster solution. The values in parentheses are the distances to the upper and lower bounds of the corresponding 95% confidence interval

In addition to the partition size, the number of clusters has an influence on the performance of both algorithms. To take a closer look at this influence we took the original IFD with dataset size of 150 and again drew partitions from a DPGMM. This time, however, we varied the concentration parameter *α* and used values of 1, 50000, 100000, 150000, 200000 and 300000. This procedure has the effect that the samples from the DPGMM tend to have a higher number of clusters, which is also noticeable from the aforementioned expected number of clusters $\mathbb {E}(C | \text {DP}) = \alpha \log N$ for the DP [19].

**Fig. 4:**
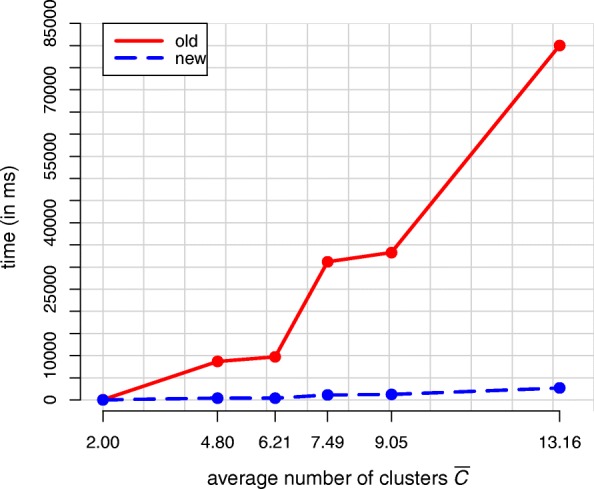
Average effort of 100 calculation repetitions using the old and the new mean partition algorithm as a function of the average number of clusters $\bar C$ within 100 sample partitions

Figure 4 presents the curve of the average effort of 100 calculation repetitions as a function of the *α*-induced average number of clusters $\bar C$ in the posterior sample. As in the last simulation, the posterior sample consisted of 100 partitions. Table 2 shows the corresponding calculation times in milliseconds, as well as the respective acceleration factor achieved by the new algorithm.

**Table 2:** Mean calculation time (in ms) of 100 calculation repetitions for different average numbers of clusters

$\bar C$	Time (old)	Time (new)	Factor
2.0	44.9 (± 0.2)	4.0 (± 0.1)	11.2
4.8	8676.6 (± 5.9)	417.3 (± 1.9)	20.8
6.21	9722.5 (± 4.2)	391.3 (± 1.5)	24.8
7.49	31186.4 (± 26.7)	1126.3 (± 2.3)	27.7
9.05	33277.8 (± 24.8)	1238.0 (± 2.2)	26.9
13.16	80026.3 (± 209.5)	2689.4 (± 8.2)	29.8

The values in parentheses are the distances to the upper and lower bounds of the corresponding 95% confidence interval

As one can see, the new algorithm proves to be much faster than the old one at both given benchmarks. This is notably true if *C*<<*N*, which is in accordance with the previously explained time complexities for both algorithms.

## Discussion

We highlighted the faster variant of the partition distance reduction of [15] and presented two further improvements of the current mean partition approximation algorithm. The first is deterministic and by at least one magnitude faster than the original method. This is the case, even if the latter makes use of the new reduction. The second theoretical enhancement employs Monte Carlo techniques and reduces the worst case complexity by almost another $\sqrt C$ to *O*(*NC*^2,575^*K*). Additionally, it also reduces the best-case runtime from originally *O*(*N*^2^*C*^3^*K*) to *O*(*NC*^1.575^*K*) per cycle. Further improvements may be possible, because the entire path to be reversed is already known before the transitive closure is updated. This knowledge could be used to calculate the dynamic transitive closure with lookahead according to [35]. Note however, that like our second improvement, this enhancement also remains theoretical at present.

## Conclusion

In this article, we have shown that the runtime of the current mean partition approximation algorithm can be significantly reduced. This makes it possible to calculate and analyze a consensus for a large set of partitions much faster, even when the number of elements and/or the number of clusters is high. We are convinced that not only the popular Structurama, which was often criticized for long runtimes, benefits from this result, but also application scenarios in which a frequent calculation of a representative partition is necessary.

## Abbreviations

APR: All-pairs-reachability
APSP: All-pairs shorthest-paths
BFS: Breath first search
DFS: Depth first search
DP: Dirichlet process
DPGMM: Dirichlet process gaussian mixture model
DPMM: Dirichet process mixture model
LSAP: Linear sum assignment problem
MCMC: Markov chain monte carlo

## References

[1] Huelsenbeck JP, Andolfatto P (2007). Inference of Population Structure Under a Dirichlet Process Model. Genetics.

[2] Huelsenbeck JP, Andolfatto P, Huelsenbeck ET (2011). Structurama: Bayesian Inference of Population Structure. Evol Bioinformatics Online.

[3] Miller JW, Harrison MT (2014). Inconsistency of Pitman-Yor Process Mixtures for the Number of Components. J Mach Learn Res.

[4] Shringarpure S, Won D, Xing EP (2011). StructHDP: automatic inference of number of clusters and population structure from admixed genotype data. Bioinformatics.

[5] Lawson DJ. Populations in statistical genetic modelling and inference. ArXiv e-prints. 2013:1306:arXiv:1306.0701. https://arxiv.org/abs/1306.0701.

[6] Ailon N, Charikar M, Newman A (2008). Aggregating Inconsistent Information: Ranking and Clustering. J ACM.

[7] Blei DM, Jordan MI. Variational methods for the Dirichlet process. New York: ACM; 2004. pp. 1–8.

[8] Goder Andrey, Filkov Vladimir (2008). Consensus Clustering Algorithms: Comparison and Refinement. 2008 Proceedings of the Tenth Workshop on Algorithm Engineering and Experiments (ALENEX).

[9] Gionis A, Mannila H, Tsaparas P (2007). Clustering Aggregation. ACM Trans Knowl Discov Data.

[10] Bertolacci M, Wirth A (2007). Are approximation algorithms for consensus clustering worthwhile?. Proceedings of the 2007 SIAM International Conference on Data Mining. Proceedings.

[11] Vega-Pons S, Ruiz-Shulcloper J (2011). A survey of clustering ensemble algorithms. Int J Pattern Recognit Artif Intell.

[12] Onogi A, Nurimoto M, Morita M (2011). Characterization of a Bayesian genetic clustering algorithm based on a Dirichlet process prior and comparison among Bayesian clustering methods. BMC Bioinformatics.

[13] Almudevar A, Field C (1999). Estimation of Single-Generation Sibling Relationships Based on DNA Markers. J Agric Biol Environ Stat.

[14] Gusfield D (2002). Partition-distance: A problem and class of perfect graphs arising in clustering. Inf Process Lett.

[15] Konovalov DA, Litow B, Bajema N (2005). Partition-distance via the assignment problem. Bioinformatics.

[16] Kuhn HW (1955). The Hungarian method for the assignment problem. Nav Res Logist Q.

[17] Munkres J (1957). Algorithms for the Assignment and Transportation Problems. J Soc Ind Appl Math.

[18] Glassen T (2018). Psychologisch orientierte Kategorisierung in der kognitiven Robotik mit dem Hierarchischen Dirichlet Prozess [Dissertation].

[19] Wallach HM, Jensen ST, Dicker L, Heller KA. An Alternative Prior Process for Nonparametric Bayesian Clustering. arXiv:08010461 [math, stat]. 2008. ArXiv: 0801.0461. https://arxiv.org/abs/0801.0461.

[20] Suri S. Bipartite Matching & the Hungarian Method. 2006. http://athena.nitc.ac.in/~kmurali/Courses/CombAlg2014/suri.pdf. Accessed 24 Dec 2017.

[21] Burkard R, Dell’Amico M, Martello S (2009). Assignment Problems.

[22] Thorup M (2004). Fully-Dynamic All-Pairs Shortest Paths: Faster and Allowing Negative Cycles.

[23] Su J, Zhu Q, Wei H, Yu JX (2017). Reachability Querying: Can It Be Even Faster. IEEE Trans Knowl Data Eng.

[24] Sankowski P (2004). Dynamic transitive closure via dynamic matrix inverse: extended abstract. 45th Annual IEEE Symposium on Foundations of Computer Science.

[25] Bird Richard S. (2006). Improving Saddleback Search: A Lesson in Algorithm Design. Lecture Notes in Computer Science.

[26] Coppersmith D, Winograd S (1990). Matrix multiplication via arithmetic progressions. J Symb Comput.

[27] Robinson S (2005). Toward an Optimal Algorithm for Matrix Multiplication. SIAM News.

[28] Gall FL. Faster Algorithms for Rectangular Matrix Multiplication. In: 2012 IEEE 53rd Annual Symposium on Foundations of Computer Science: 2012. p. 514–523.

[29] Huang J, Smith TM, Henry GM (2016). Geijn RAvd. Strassen’s Algorithm Reloaded. SC16: International Conference for High Performance Computing, Networking, Storage and Analysis.

[30] Stern KL. Hungarian Algorithm. 2012. https://github.com/KevinStern/software-and-algorithms/blob/master/src/main/java/blogspot/software_and_algorithms/stern_library/optimization/HungarianAlgorithm.java. Accessed 24 Dec 2017.

[31] Butler K, Field C, Herbinger CM, Smith BR (2004). Accuracy, efficiency and robustness of four algorithms allowing full sibship reconstruction from DNA marker data. Mol Ecol.

[32] Konovalov DA, Manning C, Henshaw MT (2004). Kingroup: a program for pedigree relationship reconstruction and kin group assignments using genetic markers. Mol Ecol Notes.

[33] Glassen TJ. Mean Partition. 2018. https://github.com/t-glassen/mean_partition. Accessed 25 July 2018.

[34] Fisher RA (1936). The Use of Multiple Measurements in Taxonomic Problems. Ann Eugenics.

[35] Sankowski P, Mucha M (2010). Fast Dynamic Transitive Closure with Lookahead. Algorithmica.

